# Improving the normalization of complex interventions: measure development based on normalization process theory (NoMAD): study protocol

**DOI:** 10.1186/1748-5908-8-43

**Published:** 2013-04-11

**Authors:** Tracy L Finch, Tim Rapley, Melissa Girling, Frances S Mair, Elizabeth Murray, Shaun Treweek, Elaine McColl, Ian Nicholas Steen, Carl R May

**Affiliations:** 1 Institute of Health and Society, Newcastle University, Baddiley-Clark Building, Richardson Road, Newcastle-upon-Tyne NE2 4AX, UK; 2 Institute of Health and WellBeing, University of Glasgow, 1 Horselethill Road, Glasgow, G12 9LX, UK; 3 Research Department of Primary Care and Population Health, University College London, Upper Floor 3, Royal Free Hospital, Rowland Hill Street, London, NW3 2PF, UK; 4 Health Services Research Unit, University of Aberdeen, 3rd Floor, Health Sciences Building, Foresterhill, Aberdeen, AB25 2ZD, UK; 5 Faculty of Health Sciences, Building 67, University of Southampton, Highfield, Southampton, SO17 1BJ, UK

**Keywords:** Normalization process theory, NPT, Implementation process, Survey, Instrument development, Complex interventions

## Abstract

**Background:**

Understanding implementation processes is key to ensuring that complex interventions in healthcare are taken up in practice and thus maximize intended benefits for service provision and (ultimately) care to patients. Normalization Process Theory (NPT) provides a framework for understanding how a new intervention becomes part of normal practice. This study aims to develop and validate simple generic tools derived from NPT, to be used to improve the implementation of complex healthcare interventions.

**Objectives:**

The objectives of this study are to: develop a set of NPT-based measures and formatively evaluate their use for identifying implementation problems and monitoring progress; conduct preliminary evaluation of these measures across a range of interventions and contexts, and identify factors that affect this process; explore the utility of these measures for predicting outcomes; and develop an online users’ manual for the measures.

**Methods:**

A combination of qualitative (workshops, item development, user feedback, cognitive interviews) and quantitative (survey) methods will be used to develop NPT measures, and test the utility of the measures in six healthcare intervention settings.

**Discussion:**

The measures developed in the study will be available for use by those involved in planning, implementing, and evaluating complex interventions in healthcare and have the potential to enhance the chances of their implementation, leading to sustained changes in working practices.

## Background

In the social sciences, implementation theory is growing in importance for policy and practice, as agencies in the public and private sector continue to foster innovation and change, and to embed and integrate organizational and technological innovations in practice. In healthcare, such innovations are diverse in scope. At times, technological advances and increasing practice-based knowledge lead to the development and assessment of particular therapeutic interventions that, if proven effective, are promoted for uptake in clinical practice. Other healthcare innovations are broader in scope, and include, for example, efforts to transform professional behaviour in line with evidence-based medicine [1], the introduction of telehealth or telecare systems attempts to overcome inefficiencies by improving access to expertise across spatial boundaries [2], and organizationally-focused service redesign initiatives that aim to increase professional governance [3]. What these types of innovations have in common is that they are intended to change practices in ways that are intended to lead to benefits and outcomes in terms of quality of care and/or efficiencies in organization.

We know already that innovations in healthcare are complex. There is a vast literature on implementation in this context, however efforts at implementing new technologies and practices remain problematic. The gap between research evidence and practice remains wide, and concerns about the large numbers of ‘pilot’ studies of new interventions—particularly those involving new technology—that never lead to sustainable services are repeatedly expressed [4]. This problem has been explored from a number of theoretical perspectives, including those that emphasize attitudes and behaviours [5]; or diffusion and adoption of innovations through social networks [6] as well as approaches from Science and Technology Studies (STS) [7] that emphasize technology design and its relations with human actors. These perspectives have offered important insights but have been difficult to apply prospectively and quantitatively [8]. Also, there can be a tendency to over-emphasize the personal agency of individuals and underplay the importance of organizational context. For example, implementation failures are often attributed to slow behavior change by individual professionals, when there are likely to be other good and predictable socio-organizational reasons for such failure [9]. Furthermore, existing approaches are limited in the extent to which they offer practical ways of facilitating implementation processes in ways that lead to the embedding of new practices within contexts of use. This study will draw on an alternative theoretical approach—Normalization Process Theory (NPT)—to move beyond understanding implementation processes in order to improve implementation outcomes in the healthcare setting.

NPT [10-14] is concerned with the generative processes that underpin three core problems: implementation (bringing a practice or practices into action); embedding, (when a practice or practices may be routinely incorporated in everyday work of individuals and groups); and integration (when a practice or practices are reproduced and sustained in the social matrices of an organization or institution). In NPT, it is postulated that: practices become routinely embedded in social contexts (‘normalized’) as the result of people working, individually and collectively, to enact them; the work of enacting a practice is expressed through the operation of generative social processes; the production and reproduction of a practice requires continuous investment by agents in ensembles of action that carry forward in time and space. There are four generative processes and concomitant investments (see Table 1 for illustrative questions):

1. sense-making that promotes or inhibits the coherence of a practice to its users. These processes are energized by investments of meaning made by participants;

2. cognitive participation that promotes or inhibits users’ enrolment and legitimization of a practice. These processes are energized by investments of commitment made by participants.

3. collective action that promotes or inhibits the enacting of a practice by its users. These processes are energized by investments of effort made by participants.

4. reflexive monitoring that promotes or inhibits users’ comprehension of the effects of a practice. These processes are energized by investments in appraisal made by participants.

**Table 1:** Questions illustrative of NPT constructs


**Coherence:**	**Collective action:**
How is a practice understood by participants?	How do participants make it work?
How do they compare it with other practices?	How are their activities organised and structured?
**Cognitive participation:**	**Reflexive monitoring:**
How do participants come to take part in a practice?	How do participants evaluate a practice?
What keeps them motivated to continue taking part?	How does this change over time and what are its effects?

NPT has already been developed, tested, and refined in studies conducted across diverse settings. These have included, *inter alia*, the implementation of ehealth systems in the UK [15-17], the redesign of primary care mental health services in Victoria, Australia [18], the development and application of decision-support tools in the Mayo Clinic Health care system [19], and evidence about their utilization in the wider clinical literature [20]. Much of the work conducted to date has been about ensuring that NPT’s core constructs can be operationalized in a stable and consistent way by multiple user constituencies. This has taken the form of road testing—robustly critiquing the theory in terms of its potential for describing key processes that underpin the success or otherwise of implementation. This has involved operationalizing NPT in qualitative studies that interrogate very different social contexts. In these contexts, the criteria for stability were that the generic constructs could be translated into specific contexts without the addition of *ad hoc* conditions, and that sceptical researchers were able to use them in practice with minimal support. Exploratory work on translating NPT constructs into structured instruments has already been conducted [16,21,22]. In a previous project [23], we have undertaken work that has given us the basis for a set of generic statements for each of the constructs of NPT. This highlighted important issues concerning the framing of questions and response scales, and the challenges of multiple stakeholder assessments (and requirements for specification and tailoring as appropriate to stakeholder groups) [21]. Importantly, however, it has given us confidence that the constructs of NPT have good face validity, and that NPT has practical value to user communities for assessing and guiding the development of new interventions [24]. NPT has been shown to have excellent descriptive power; what is not yet known is its potential for use in predicting the likely outcome of an attempt to implement an intervention, and whether NPT can be used prospectively to enhance implementation. To this end, the theory warrants further critical investment in the development of instruments for quantitative research.

However, if we wish to improve the implementation of complex interventions, further work is required in two key areas. First, there is a need to develop simple quantitative tools (in the form of questionnaires) that can be easily used by those (*e.g.,* researchers, clinicians, managers, policy makers) involved in planning and undertaking implementation activities to assess and identify aspects of the implementation process that may promote and/or inhibit the likelihood of the intervention becoming sustained in practice. An important source of the evident international interest in NPT has been its potential to be used prospectively and quantitatively rather than being confined to descriptive *post hoc* analysis of specific case studies [25]. Although a small number of instruments to assess ‘readiness’ for technology-based interventions in healthcare already exists [26], they are limited in scope, and do not explicitly focus on assessing processes relevant to interventions becoming embedded in routine practice. In our previous work, we developed a simple 16-statement ‘toolkit’ containing items representing the theoretical constructs of the NPT for use as a ‘sensitizing tool’ by individuals involved in planning and implementing complex interventions to think through which aspects of their interventions might affect successful normalization of them [23]. Although developed through intensive item-development and user feedback activities, the tool was neither developed for use as a research instrument nor validated for this purpose. The second area needing further development concerns further testing of the NPT as an explanatory theory. NPT was initially developed from studies of health technology implementation in the context of clinicians and their working environments. Over recent years however, it has been developed as a middle-range theory of socio-technical change. Further development of the theory thus requires explicating features of context that are relevant to the utility of NPT for application to practice-based problems (including further understanding of ‘normalization’ as an outcome), and exploring the relationships between components of the theory to further develop understanding of the relative importance of the different processes to which NPT gives prominence in explaining how new practices become normalized.

### Aims and objectives

This study aims to operationalize the constructs of the NPT and create a simple generic instrument that can be used to enhance the implementation of complex interventions.

### Specific objectives

Our objectives are:

1. To undertake preliminary work (literature review, context mapping) to further develop a set of NPT-based measures (NPT instrument). This will inform the development of a generic instrument to be used in the evaluation of complex health interventions.

2. To evaluate the utility of the NPT instrument across a range of complex health interventions and contexts, identifying key contextual factors that may inform further refinement and or use of the tool.

3. To explore the utility of the NPT instrument for purposes of identifying, monitoring,and assessing the progression and integration of an intervention.

4. To test the adequacy of the NPT instrument as (statistical) representations of the constructs and processes as set out by the NPT.

5. To explore the predictive utility of the NPT instrument for explaining normalization outcomes.

6. To develop a comprehensive users’ manual for use in conjunction with the instrument.

## Methods

The study design consists of four phases to be undertaken over three years.

### Phase one: conceptual development and sampling framework

In our previous studies, we have focused on developing and operationalizing the four main constructs of the NPT (coherence, cognitive participation, collective action, and reflexive monitoring) and their underlying dimensions and simultaneously qualitatively testing their conceptual distinctiveness. The first phase of this study involves work to further extend theory development, looking in detail to identify important contextual features for testing out the limits of the theory and identify and critique multiple approaches to defining and measuring normalization ‘outcomes.’ This will involve a two-step process. First, we will include a review of the literature to explore the context in which NPT is being used, the range of study designs, and the application of NPT in different settings. It is anticipated that the outcome of this phase will be an inventory of NPT research to date that can be kept updated across the duration of the study. Second, using the inventory as point of reference, a contextual mapping workshop will be held to develop a contextual framework for sampling complex interventions to be used in the testing phase.

### Phase two: instrument development and refinement

The aim of phase two is to develop additional items for the existing toolkit to further represent the theoretical constructs of NPT. Most of the instrument development work will focus on items representing these constructs (process items), however a small number of measures relating to implementation outcomes (outcome items) will also be developed for the purpose of statistical analysis in phase three.

### Process items

Drawing on the expertise of the core research group, additional process items will be developed to form a preliminary instrument. This will include a process of developing a group of items that represent and build upon the original four constructs, vigorously testing them through ongoing feedback and refinement within the core research group, and systematically evaluating items utilizing, for example, the Questionnaire Appraisal System (QAS-99) [27,28] and the Question Understanding AID (QUIAD) program for assessing survey questions [29].

The validity and acceptability of the developed items will be assessed in two concurrent stages. First, a sample of 30 researchers involved in evaluating complex health interventions will be recruited to participate in cognitive interviews. Participants will be recruited through email invitation to principal investigators (PIs) leading complex health intervention studies that are listed on various publicly available research study databases and investigator contacts. Cognitive interviews techniques, such as think aloud interviewing and structured probes, which are widely used techniques in the development of surveys [30], will be used to identify and address potential problems of item comprehension and response selection. Cognitive interviews will occur in three rounds over a period of six months and will last between 20 and 30 minutes each. Qualitative analysis [31] of participants’ responses will enable us to add new items, revise existing ones, and drop those that they consider redundant or lacking face validity.

Second, to further ensure that additional items developed continue to represent theoretical concepts, we will work with members of international research groups already using NPT. Drawing on on-line survey methods used in the previous study, and for which good response rates were achieved, we will invite these users (n = 30) to provide focused feedback about the relationship between items and the theoretical concepts they are intended to represent. Participants will be asked to work through a specific problem of implementation/integration using the items, suggesting revisions to the items if appropriate. Participants will then be asked a number of questions about their experience of using the NPT items and any additional feedback about the items.

Development of the final instrument will draw on the analysis of researcher and peer feedback, item appraisal and consultation with the research group. Once a final version of the instrument is approved, an on-line version that is easily accessible and secure will be developed alongside a paper version for testing within phase three.

### Outcome items

Members of the core research team, co-applicants, and collaborators will generate a small number of ‘normalization indicators’ (n = 2 to 4) and structured statements for assessing them, that could be combined to generate a normalization ‘score.’ These are likely to consist of statements that are perception-based (*e.g.*, ‘the [intervention] has become part of our everyday working practices’), but may also utilize behavior-related data if available (*e.g.*, usage data, if the intervention is a new tool or system).

### Phase three: instrument testing

The aim of phase three is to assess the psychometric properties of the NPT instrument and provide further knowledge about the constructs of the NPT and relationships among them.

### Sample

We anticipate that a minimum of six sites will be included in the survey (on-line and paper-based), which will include participants from a variety of professional roles in relation to the intervention that is the subject of the assessment (for example, clinical, administrative, managerial, and other professionals in non-health contexts). To allow a comprehensive test of the NPT instrument, sites will be carefully chosen to ensure the inclusion of contexts that differ in important ways. The sampling framework developed in phase one will be used to guide study site collection to ensure that a maximum variation sample is achieved and to represent key contextual features. Participants in individual sites will be identified through key contacts employed in the sites and invited to participate via electronic messages.

### Data collection

At each site, surveys will be conducted using a variety of approaches (including electronic and paper-based) that are chosen to allow the best chance of maximizing response rates [32]. This will include visiting sites in person, regularly, to request survey participation (for example, by undertaking survey completion during arranged staff events if/where possible), and the use of multiple repeated electronic reminders (maximum two reminders) to encourage late responders. Inclusion of a site in the project will be conditional on the research team having direct access to potential participants’ contact details for the purpose of issuing survey invitations and reminders, which will be arranged through key contacts employed in study sites. At each site, data collection will be undertaken at multiple time-points (n = 1 to 3, depending on stage of normalization at time of recruitment into the project). Intervals between data collection points will be determined in advance, but on an individual basis for study sites, to be consistent with the observed and/or anticipated pace of normalization processes at particular sites. This will ensure that there is sufficient time between data collection points for changes to be reasonably expected. The size of the sampling population will vary greatly between sites, but a minimum pooled sample of n = 300 will be surveyed as representing an upper estimate of the sample size recommended for a reliability study [33].

### Ethics

Ethical approval for this study has been granted by the University of Newcastle Ethics Committee (Reference number 00555/2012; approval granted 1/09/2012). Informed consent will be sought from participants taking part in cognitive interviews, including having the interviews audio recorded and transcribed, and consent from participants completing the survey instrument in phase three testing. All identifying information about participants from interviews and survey completion will be removed and data analysis will consist of collated information only. All research data will be anonymized and stored electronically (password protected) on the Newcastle University network.

### Analysis: establishing validity and reliability

The psychometric properties of validity and reliability will be assessed. Analysis of item completion rates and patterns of endorsement of response categories will be used to determine those items that appear to be unacceptable/irrelevant to respondents (*i.e.*, with item completion rates less than 90%) and those which do not adequately discriminate between respondents (*i.e.*, where endorsement rates for any single response category exceed 90%). Construct validity will be explored initially through examination of the item correlations. We would expect items from the same construct to be more highly correlated with each other than with items measuring different constructs. The relationship between items will then be examined more formally using exploratory factor analysis; items loading more highly on a factor(s) other than the one they are expected through (on the basis of the theoretical model) will be eliminated. Construct validity will be further examined by ‘known group’ tests, using statistical techniques (*e.g.*, t-tests, ANOVA) appropriate to the distribution of scores and to the nature of the classifying variable, scores for each domain of the instrument will be compared across groups hypothesized, on the basis of the theoretical model of normalization, to differ from one another (*e.g.*, between groups of individuals who have and have not integrated an intervention successfully). Two aspects of instrument reliability will be addressed. Internal consistency will be measured through the statistics of item-total correlations and Cronbach’s alpha. A subset of respondents (n = 50) will be asked to complete the instrument on a second occasion between two and four weeks after the initial administration (a period over which change in normalization is not expected in that particular context); test-retest reliability will be assessed through intra-class correlations.

### Testing the propositions of the NPT

Analysis of survey data will focus on answering the following questions:

1. Does the NPT instrument detect change over time in responses to items representing NPT constructs?

2. Does change in these items relate to change in responses to items representing normalization indicators?

Regression analyses will be conducted with the measures of normalization (developed in phase two) as the dependent variable and NPT construct scores as independent variables, with contextual factors included as covariates. For sites where the construct measures have been undertaken at multiple time-points with individual participants, repeated measures analysis would be undertaken.

### Phase four: prepare ‘user manual’ for quantitative assessment of NPT

The analyses of phase three will generate useful knowledge about how to apply and understand the results of using the generic NPT instrument in a range of contexts in which new practices are to be assessed. In relation to other theories (*e.g.*, Theory of Planned Behavior [34]), accessible manuals for guiding the design and conduct of quantitative surveys have proven popular [35]. In this phase, we will bring together the generic instrument and the knowledge gained from all phases of the project to produce a simple manual for guiding quantitative assessments based on the NPT. This will be linked into the NPT Online Users Manual that we have already developed in our previous study (http://www.normalizationprocess.org).

## Discussion

The project described in this paper builds on an ongoing program of research and user engagement focused on developing theory that has applied relevance to practical problems of implementation of health-related interventions (achieved through engagement with relevant user communities), and making the theory (NPT) itself widely accessible to diverse communities of users (academic, policy, managerial, clinical) in the form of tools to guide implementation and facilitate evaluation.

The potential for NPT to have far-reaching impact on academic and applied activity to improve the development of complex interventions that are well-placed to become effectively normalized in practice is evident, as demonstrated by the increasing volume of published research that has utilized NPT as a framework for evaluation studies [36-54]. However, achieving this impact requires more sophisticated (but simply administered) assessment measures to be developed, tested, and made available to user groups.

The project thus also aims to build on and extend previous engagement with diverse user communities with interests in improving the implementation of complex interventions. We have already developed and made available an online NPT ‘Users Manual’ (http://www.normalizationprocess.org) that includes explanatory tools that can support and facilitate developments in practice and policy settings. However, the toolkit [23] is designed specifically for academic and non-academic users of NPT to think through their implementation problems, almost as a ‘sensitizing’ device rather than as a validated tool for measuring implementation process and outcomes (as is the objective of this project). In this project, wider engagement activities will be undertaken, including workshops to draw on the expertise and knowledge of user groups outside the project team (including those who are not familiar with NPT or have not previously used applied it in their work), and the inclusion of (as data collection sites) approximately six major study sites that will participate in instrument testing. These sites will be presented with summarized data reflecting assessments undertaken regarding the interventions they are implementing, and have opportunity to feedback about the utility of this information that will be important for ongoing development of the measures and for informing their application. Representatives from study sites will also be invited to participate in a launch event planned for the conclusion of the project, as will others contributing to the study.

The primary output of the study, however, will be the NPT measures that are to be developed in this project, along with validation data concerning statistical properties and other information that can be used to guide the application of the measures across different settings and for different purposes (designing, monitoring, trouble-shooting interventions and their implementation). We anticipate that although the measures themselves will be an important product of the study, the generation (and dissemination) of experience and knowledge in the application of these measures to real problems of implementation of complex health interventions in diverse settings will be the key to improving the design and implementation of interventions that are ultimately intended to benefit recipients of health care services.

## Competing interests

The authors declare that they have no competing interests.

## Authors’ contributions

All authors have made substantial contributions to the study conception and design, and the development and editing of the manuscript. TF and CRM led initial study design and development and wrote the original study protocol with conceptual and design contributions from all authors. TF, TR and MG further refined the study protocol and drafted the publication with contributions from all authors. All authors are contributing to the conduct of the study and have read and approved the manuscript for publication.
